# Medicines Prices and Malaysia—Untangling the Medicines Web

**DOI:** 10.1371/journal.pmed.0040149

**Published:** 2007-03-27

**Authors:** Suzanne Hill

## Abstract

Hill discusses the implications of a new survey on drug prices and availability in Malaysia.

Pharmaceuticals make a major to contribution to health. However, as noted by Ess et al., in Europe over the last 20 years, expenditure on pharmaceuticals has grown faster than the gross national product, and Wagner and McCarthy described the problem in low-income countries where “the vast majority are unwilling to pay for effective drugs simply because they are unable to pay” [1,2]. Up until the 1990s, studies examining drug prices were few and far between, and in developing countries were nonexistent. Measuring comparative prices of medicines was seen as complex, and rightly so—many methodological challenges face such studies (Box 1) [3,4].

Work by Health Action International (HAI) in the late 1990s started to address these questions. In 1998, a study reported by HAI [5] described the variability of the price of ranitidine, sampled in multiple countries. In India at that time, one hundred 150 mg tablets could be bought for USD 2, whereas in South Africa, the price was USD 150. The key message from this study was that prices of common medicines vary enormously between countries and often within countries as well. If affordability is a barrier to availability of medicines, this was one of the first studies to try to quantify the gap.

## A New Study on Drug Pricing

In a new study published in *PLoS Medicine*, Babar et al. [6] report the results of a survey of drug prices and availability in Malaysia. This is one of many surveys that have been carried out, using a standard methodology for pricing surveys that has been developed by HAI in collaboration with the World Health Organisation since 1998. This standard method encourages systematic data collection, reporting, and analysis of prices of a “basket of medicines” to enable comparison within and across countries with the aim of informing pharmaceutical policy development. The method has been field tested, validated, and used in nearly 50 surveys in different countries, the results of which are all publicly available on the HAI Web site (http://www.haiweb.org/medicineprices). Having an internationally agreed method for surveys is an important step in improving the acceptance of the results; medicine prices are always a politically sensitive subject.

**Box 1. Methodological Challenges in Studying Comparative Drug Prices**

- What products do you sample?
- Where do you sample?
- If you choose to sample retail suppliers, in what area do you sample them?
- How many samples from each source?
- Brand or generic medicines?
- What if there are multiple generic versions of the same medicine?
- How do you measure the components that contribute to the final price paid by the patient/consumer?
- How do you compare across countries, given very different systems of supply and pricing structures?

In the Malaysian context, which is a “free market” for medicines, the authors documented significant variation in the prices of the standard sample of medicines. In the private sector, prices were substantially higher than the international reference price. Where a doctor was allowed to dispense medicines as well as prescribe them, there was a similar discrepancy in price, with very high markups being charged. Markups at other points in the pharmaceutical supply chain were also found to be high, contributing to high drug prices overall. For a selected subsample of medicines, detailed data were collected on all components of the final prices: the markups added along the supply chain from the manufacturer to the point of sale to the patient. Based on the index measure of affordability—a day's wages for a government worker—the authors suggest that even in the public sector, many essential medicines would be unaffordable. The overall assessment was that compared to international reference standards and other countries at the same level of development, Malaysian medicine prices were very high.

## Limitations of the Study

The limitations of this study are those of most one-off cross-sectional surveys: How representative are the results for the pharmacies and facilities sampled overall? In a country of 20 million people with hundreds of hospitals and pharmacies, is it sufficient to draw general/national conclusions from a sample of 20 public hospitals, 32 private sector pharmacies, and 20 dispensing doctors? This is clearly an important limitation, and ideally repeating the survey using a larger sample, or with repeated measures over time, would provide a more robust picture. However, in the absence of automated systems for recording the dispensing, prescribing and cost of medicines, data collection of medicine prices is labour intensive, and it is therefore necessary to balance the ideal with the possible. Having some initial results provides a starting point for untangling the problem of high drug prices in Malaysia.

## Policy Implications

The challenge now for the authors, and also for the HAI project overall, is to answer the “so-what” question. Prices of medicines in Malaysia may be high, but are these prices really affecting access to medicines (hard to measure) or health outcomes (even harder to measure)? Should there be wholesale policy change from a “free market” to control of medicine prices? If so, should all points in the supply chain be regulated—that is, from manufacturer to dispensing—or just some? If so, how? What policies are possible, and what impact would they have? South Africa has taken the step of introducing controls on pharmacist dispensing fees [7], but could have also opted to regulate other parts of the supply chain—what would be the impact? In a low- or middle-income country, how do you decide on the tradeoff between regulating prices and supporting the local pharmaceutical industry, which might be your main source of cheap(er), but good quality products? A systematic review by Aaserud et al. [8] published last year summarized studies of some pricing policies that are widely used in developed countries, and concluded that there is very little information to assess benefits and harms of particular policy options, in terms of impact on prices, affordability, or health comes. But this review did not find any studies from developing countries that could inform decision-making, and most of the policies were variations on reference pricing approaches.

## Next Steps

As noted on the HAI Web site, “Reliable data is the first step to exploring policy options and taking action.” There are many question to answer. For each country that has done the survey, like the Malaysian study, the first question is whether this a true picture of the situation and whether it remains so over time. The study samples only a limited number of medicines—what about other important groups (such as children and the terminally ill)? But most importantly, once the data are collected, what are the policy options for a country and what impact will they have? If there is to be any chance of evidence informing policy decisions in this challenging field, then the transparent process encouraged and developed by HAI must continue—prices cannot be the subject of closed-door negotiations and the factors that contribute to pricing should be detailed so that those who pay know what they are getting for their money.
